# WGCNA Reveals Hub Genes and Key Gene Regulatory Pathways of the Response of Soybean to Infection by *Soybean mosaic virus*

**DOI:** 10.3390/genes15050566

**Published:** 2024-04-27

**Authors:** Jingping Niu, Jing Zhao, Qian Guo, Hanyue Zhang, Aiqin Yue, Jinzhong Zhao, Congcong Yin, Min Wang, Weijun Du

**Affiliations:** 1 College of Life Sciences, Shanxi Agricultural University, Taigu, Jinzhong 030801, China; niujingping@sxau.edu.cn; 2 College of Agronomy, Shanxi Agricultural University, Taigu, Jinzhong 030801, China; z15517796474@163.com (J.Z.); 17798242471@163.com (Q.G.); 15204216452@163.com (H.Z.); yueaiqinnd@126.com (A.Y.); wangmin3502@126.com (M.W.); 3 Department of Basic Sciences, Shanxi Agricultural University, Taigu, Jinzhong 030801, China; zhaojz@sxau.edu.cn (J.Z.); sxndycc@163.com (C.Y.)

**Keywords:** soybean, SMV, RNA-Seq, WGCNA, regulatory pathway, hub genes, RT-qPCR

## Abstract

*Soybean mosaic virus* (SMV) is one of the main pathogens that can negatively affect soybean production and quality. To study the gene regulatory network of soybeans in response to SMV SC15, the resistant line X149 and susceptible line X97 were subjected to transcriptome analysis at 0, 2, 8, 12, 24, and 48 h post-inoculation (hpi). Differential expression analysis revealed that 10,190 differentially expressed genes (DEGs) responded to SC15 infection. Weighted gene co-expression network analysis (WGCNA) was performed to identify highly related resistance gene modules; in total, eight modules, including 2256 DEGs, were identified. Kyoto Encyclopedia of Genes and Genomes (KEGG) pathway enrichment analysis of 2256 DEGs revealed that the genes significantly clustered into resistance-related pathways, such as the plant–pathogen interaction pathway, mitogen-activated protein kinases (MAPK) signaling pathway, and plant hormone signal transduction pathway. Among these pathways, we found that the flg22, Ca^2+^, hydrogen peroxide (H_2_O_2_), and abscisic acid (ABA) regulatory pathways were fully covered by 36 DEGs. Among the 36 DEGs, the gene *Glyma.01G225100* (protein phosphatase 2C, PP2C) in the ABA regulatory pathway, the gene *Glyma.16G031900* (WRKY transcription factor 22, WRKY22) in Ca^2+^ and H_2_O_2_ regulatory pathways, and the gene *Glyma.04G175300* (calcium-dependent protein kinase, CDPK) in Ca^2+^ regulatory pathways were highly connected hub genes. These results indicate that the resistance of X149 to SC15 may depend on the positive regulation of flg22, Ca^2+^, H_2_O_2_, and ABA regulatory pathways. Our study further showed that superoxide dismutase (SOD) activity, H_2_O_2_ content, and catalase (CAT) and peroxidase (POD) activities were significantly up-regulated in the resistant line X149 compared with those in 0 hpi. This finding indicates that the H_2_O_2_ regulatory pathway might be dependent on flg22- and Ca^2+^-pathway-induced ROS generation. In addition, two hub genes, *Glyma.07G190100 (*encoding F-box protein) and *Glyma.12G185400* (encoding calmodulin-like proteins, CMLs), were also identified and they could positively regulate X149 resistance. This study provides pathways for further investigation of SMV resistance mechanisms in soybean.

## 1. Introduction

SMV disease, caused by the aphid-transmitted and seed-borne SMV from the genus *Potyvirus* and the family *Potyviridae*, is a serious yield-suppressing disease [1,2,3]. The disease was first found in the United States as early as 1915, after which it appeared in soybean-growing countries, including Brazil, Canada, China, Japan, and South Korea, as well as the United States [4]. Currently, many SMV strains have been identified and classified according to disease symptoms in soybean differentials; 5 strains (A–E) were identified in Japan [5], 98 isolates were classified into 7 strains (G1-G7) in North America [6], and more than 4500 isolates were collected and classified into 22 strains (SC1-SC22) in China [7,8,9,10,11,12,13].

Based on the interaction between viruses and plants, the types of plant resistance to viruses are thought to include dominant resistance and recessive resistance [14,15]. To our knowledge, approximately 34 single dominant resistance loci for SMV located on six chromosomes have been identified and reported in soybean: chromosome 02 (*Rsv4*, *RSC5*, *RSC6*, *RSC7*, *RSC8*, *RSC10*, *RSC17*, *RSC18A*, *RSC3(w)*, *RkSC13*, *Rsc13*, *RSC9*, and *Rsc3k*) [16,17,18,19,20,21], chromosome 03 (*qTsmv-13*) [22], chromosome 06 (*RSC15*, *RSC18*) [2,20], chromosome 11(*RSMV-11*) [23], chromosome 13 (*Rsv1*, *Rsv5*, *RSC3Q*, *RSC11*, *RSC12*, *RSC14Q*, *RSC18B*, *RSC20*, *RSC-pm*, *RSC-ps*, *Rsv1-r*, *RSC15ZH*, *qSMV13*, and *Rsvg2*) [20,24,25,26,27,28], and chromosome 14 (*Rsv3*, *RSC4*, and *rySC13*) [20,29]. Among the above five resistance loci, for which gene cloning and functional analysis have been completed, *Glyma.14g204700* on *Rsv3* [30], *Rsc4-3* on *Rsc4* [31,32], *GmCAL* on *Rsc8* [33], *GmST1* on *Rsvg2* [28], and a recombinant gene on *Rsc3k* are involved in soybean resistance to SMV [17].

Resistance genes have also been screened by RNA sequencing (RNA-Seq) technology. The expression of transcripts related to pathogen recognition, autophagy, and defense inductors was up-regulated upon SMV infection [34]. The involvement of genes related to ABA, siRNA, and autophagy pathways in L29 *Rsv3*-mediated resistance to SMV strain G5H was indicated by transcriptome analysis [35]. The transcriptome analysis revealed a down-regulation of NBS-LRR family genes in the susceptible variety Hefeng25 when infected with SMV N1 [36]. Potential genes related to ABA, plant–pathogen interaction, and jasmonic acid (JA) pathways related to *Rsc3Q*-mediated resistance to SMV strain SC3 were identified via transcriptome analysis between the R (*RSC3Q*) and S (*rSC3Q*) lines from the cross Qihuang-1 (which carries the resistance gene *RSC3Q*) × Nannong1138-2 (which carries the susceptible gene *rSC3Q*) [37]. Transcriptome analysis of the response of the resistant variety Kefeng-1 to SMV strain SC18 and the susceptible variety NN1138-2 also revealed that several genes related to the ethylene (ETH), jasmonic acid (JA), and salicylic acid (SA) signaling pathways may be involved in the disease resistance to SC18 [38]. In addition, the transcriptome analysis also revealed the influence of genes involved in Ca^2+^ regulatory and MAPK cascade pathways on SC15 resistance in the resistant variety Kefeng-1 [39]. In short, the aforementioned genes were acquired through conventional transcriptome analysis and are predominantly situated within the pathways of plant–pathogen interaction and plant hormone signal transduction pathways. The only exception is that one of the two pathways for Ca^2+^ regulation is a complete pathway, while the others are incomplete [39].

The WGCNA method, which is based on conventional transcriptome analysis, has been widely and successfully employed in the field of bioinformatics to identify the relationship between module eigengenes and hub genes with sample traits [40]. Two modules and seven hub genes were identified as being associated with the resistance of pepper to *pepper mild mottle virus* [41]. The three pathways (MAPK signaling pathway, plant–pathogen interaction, and glutathione metabolism) and 14 hub genes were identified based on the modules’ highest correlation with eggplant bacterial wilt-resistance [42]. To date, WGCNA study has not yet been reported on soybean response to SMV infection.

The present study aimed to investigate gene co-expression networks associated with SC15 resistance in soybeans. Firstly, the expression of DEGs between resistance line X149 and susceptible line X97 was analyzed at the transcriptional level. Secondly, WGCNA was performed based on DEGs, and target gene modules associated with SC15 resistance were screened. Thirdly, the KEGG pathway enrichment analysis was conducted to reveal the key resistance-related regulatory pathways in the target gene modules. The hub genes in the target gene modules were identified according to the connectivity of genes in the co-expression network. Lastly, oxidase activity and H_2_O_2_ content measurements in resistant line X149 were used to explore the response of soybean to SC15 infection. These findings have important theoretical significance for revealing the molecular mechanism of soybean to SMV and provide new genetic resources for breeding of SMV resistance in soybean.

## 2. Materials and Methods

### 2.1. Plant Materials, SC15 Inoculation, and Detection of SMV Virus

The soybean resistant line X149 and susceptible line X97, obtained from the Soybean Germplasm Innovation and Utilization Laboratory of Shanxi Agricultural University in 2019, had been used to map resistance loci by genome-wide association mapping (GWAS) [43]. The SC15 strain was provided by Professor Haijian Zhi of Nanjing Agricultural University.

X149 and X97 were planted in 14 cm diameter plastic pots containing nutrient soil and vermiculite (volume ratio 1:2). A total of 15–20 seedlings were planted in each pot, and the seedlings were subsequently placed in a greenhouse at 25 °C with a 16 h light/8 h dark photoperiod. In total, three pots were planted, and one pot represented one biological replicate. The fully expanded primary leaves were inoculated with SC15 using the gentle rubbing method [2]. Approximately four SC15-infected leaves were collected at 0, 2, 8, 12, 24, and 48 h post-inoculation (hpi), and three replicates were performed. These 36 samples were used for RNA-Seq.

Further, X149 and X97 were also planted and inoculated with SC15 according to the method mentioned above for SC15 detection by real-time quantitative PCR (RT-qPCR). After 20 days post-inoculation, leaves of necrotic symptoms in X97 and of no symptoms in X149 were collected. Three replicates were performed, with X149 and X97 inoculated with phosphate-buffered saline (PBS, 0.01 M, pH 7.4) as controls. Total RNA was isolated and extracted using the EZ-10 DNAaway RNA Mini-Preps Kit (Sangon Biotech, Shanghai, China) according to the manufacturer’s protocol. Subsequently, SC15 cDNA synthesis was performed using the GoScript^TM^ Reverse Transcription System (Promega, Beijing, China) commercial kit as per the manufacturer’s instructions. The CP genome sequence of SC15 was obtained from GenBank (MH919386.1) and primers for RT-qPCR were designed by Primer Premier 5.0. The volume and program settings for RT-qPCR were adjusted according to the specifications of MomAmpTM Chemohs qPCR (Monad Biotech, Suzhou, China). RT-qPCR was subsequently conducted using a Bio-Rad CFX96 system. Primer information of SC15 CP is shown in Table S1.

### 2.2. RNA Extraction, Library Preparation, and Sequencing

A total of 36 RNA samples were extracted with a TRIzol^®^ reagent (Invitrogen, Carlsbad, CA, USA) following the manufacturer’s instructions. The qualitative and quantitative assessments of the RNA were detected using a Nanodrop2000 (NanoDrop Technologies, Thermo Fisher Scientific, Waltham, MA, USA). The deterioration and contamination of RNA was detected using 1% agarose gel electrophoresis [44], and RNA integrity (RIN) was determined with an 2100 Bioanalyzer (Agilent Technologies, Santa Clara, CA, USA). Finally, only RNA samples that met the following criteria were used to construct the sequencing library: OD260/280 =1.8~2.2, OD 260/230 ≥ 2.0, and RIN ≥ 7.0 [45]. The RNA-seq transcriptome libraries of 36 samples were prepared at Shanghai Majorbio Biopharm Biotechnology Co., Ltd. (Shanghai, China) following the manufacturer’s instructions protocol (Illumina, San Diego, CA, USA) and sequenced using the Illumina NovaSeq 6000 platform.

### 2.3. Analysis of RNA-Seq Data

Clean reads from each sample were obtained after the raw reads were filtered with fastp [46]. The mapped reads of each sample were evaluated by clean reads alignment of the reference genome willimas82. a4 (http://phytozome.jgi.doe.gov/pz/portal.html (accessed on 10 November 2021)) with HISAT 2. 2.1 software [47]. All the mapped gene functional annotations are described by six databases: Non-Redundant Protein Sequence Database (NR) (ftp://ftp.ncbi.nlm.nih.gov/blast/db/ (accessed on 10 November 2021)) [48], Swiss-Prot (http://web.expasy.org/docs/swiss-prot_guideline.html (accessed on 10 November 2021)) [49], Pfam (http://pfam.xfam.org/ (accessed on 10 November 2021)) [50], Evolutionary Genealogy of Genes: Non-supervised Orthologous Groups (EggNOG) (http://www.ncbi.nlm.nih.gov/COG/ (accessed on 10 November 2021)) [51], Gene Ontology (GO) (http://www.geneontology.org (accessed on 10 November 2021)) [52], and KEGG (http://www.genome.jp/kegg/ (accessed on 10 November 2021)) [53]. The gene abundance of each transcript was quantified with RSEM v1.3.3 software (http://deweylab.github.io/RSEM/ (accessed on 15 November 2021)) [54]. The quantitative index was the number of fragments per kilobase per million reads (FPKM). The differential expression genes (DEGs) between different transcripts were identified using the DESeq2 R package 1.42.0 [55,56]. Genes with a fold change ≥2 and FDR adjusted *p*-value < 0.05 were considered significant DEGs. Upset diagrams were constructed on the online tool of the Majorbio cloud platform (https://cloud.majorbio.com/page/tools/ (accessed on 15 November 2021)) [57].

### 2.4. Gene Co-Expression Network Analysis

WGCNA was performed using the Majorbio cloud platform [57]. To improve the accuracy of the WGCNA, genes with low expression (FPKM < 1) were removed. The modules were identified using the parameter soft threshold power (β value), the minimum value of cluster size was 30, and the merging threshold was 0.25. Determination of the β value was based on the adjacency matrix using WGCNA. The adjacency matrix was weighted by the power of correlation data between different genes, and the β value was determined from the scale-free topology and average connectivity criterion [58]. The modules correlating with resistance were calculated and *p* < 0.01 was used as the relation module screening criterion. The correlation coefficient between the modules and resistance was positive for positive correlations and negative for negative correlations. The visual co-expression network was constructed using the Cytoscape [59], representing the top 30 hub genes of modules correlating with resistance. Enrichment analysis of the KEGG pathways associated with the modules was carried out by KOBAS [60].

### 2.5. RT-qPCR Analysis

The template cDNA of the RNA-Seq samples was synthesized using the MonScriptTM RTIII Super Mix with dsDNase (Monad Biotech., Suzhou, China) following a standard protocol. The soybean CDS for verification genes were obtained from the Phytozome database. The gene primers for RT-qPCR were designed by Primer Premier 5.0. The housekeeping gene Tubulin [61] was employed as a reference control in RT-qPCR analysis of RNA-Seq samples. The volume and program settings for RT-qPCR were adjusted according to the specifications of MomAmpTM Chemohs qPCR (Monad Biotech., Suzhou, China). RT-qPCR was subsequently conducted using a Bio-Rad CFX96 system. Three technological replicates were conducted for each sample, and the relative expression of four genes was calculated with the relative 2^−ΔCT^ method. Information on the primers used is shown In Table S1.

### 2.6. SOD, CAT, POD Activity, and H_2_O_2_ Content Assays

The SC15-infected leaves of X149 were collected at 0, 2, 8, 12, 24, and 48 hpi, and three replicates were performed. Subsequently, SOD, CAT, and POD activities were detected using Nanjing Jiancheng Bioengineering Institute (NJBI) kits (A001-1, A007-1, and A084-3; NJBI, Nanjing, China) following the manufacturer’s instructions. The H_2_O_2_ content was detected using a Beijing boxbio determination kit (AKAO009C, Boxbio, Beijing, China) following the manufacturer’s instructions. The instrument used for enzyme activity determination was an ultraviolet-visible spectrophotometer (UV-1100, Molecular Devices., Tianjin, China). 

## 3. Results

### 3.1. Detection of SMV Virus in Soybean Lines

**Figure 1:**
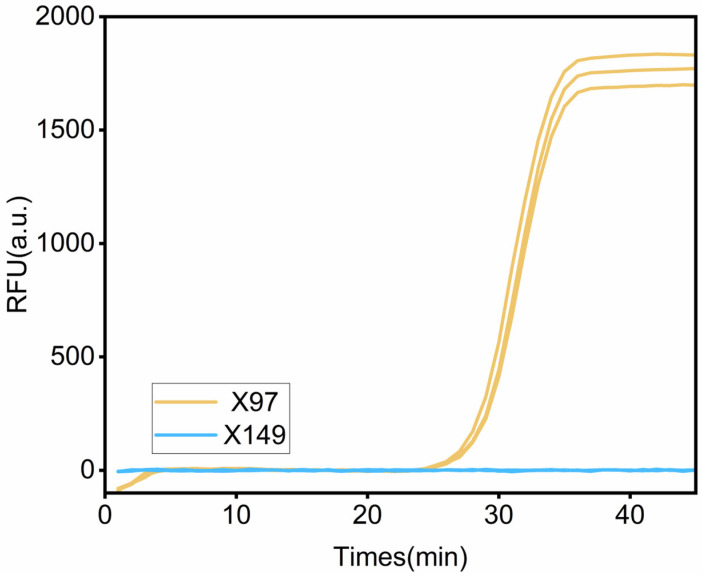
Real-time fluorescence amplification curves of SMV SC15 in resistant line X149 inoculated with SC15 and susceptible line X97 inoculated with SC15.

The leaf phenotype of X149 inoculated with SC15, X149 inoculated with PBS, and X97 inoculated with PBS exhibited no symptoms after inoculation for twenty days, whereas the leaf phenotype of soybean line X97 inoculated with SC15 displayed necrotic symptoms. We employed SC15 CP primer to detect the presence of SC15 in both X149 and X97 leaves using RT-qPCR. The results revealed that amplification was not observed in X149 inoculated with SC15 (Figure 1), X149 inoculated with PBS, and X97 inoculated with PBS, while normal amplification was observed in X97 inoculated with SC15 (Figure 1). These findings indicate that necrotic symptoms were caused by SC15 infection.

### 3.2. Transcriptome Sequencing Data Analysis

To analyze the genes involved in the response to SC15 resistance in soybeans, transcriptome sequencing was carried out with the resistant line X149 and susceptible line X97 at six time points (0, 2, 8, 12, 24, and 48 hpi). Approximately 45,531,879 clean reads were generated on average (Table S2). The Q20 and Q30 percentages of each sample were more than 97.97% and 94.10%, respectively (Table S2). The average GC content of all the samples was 45.33% (Table S2). An average of 93.38% of the clean reads were mapped to the reference genome, and 86.64% were uniquely mapped (Table S2). A total of 52,872 unique genes were described, and the expression levels were quantified according to the expected FPKM values. Among these, 24,636 DEGs with a fold change ≥ 2 and FDR-adjusted *p*-value < 0.05 were screened and considered for further analysis.

### 3.3. Identification of DEGs

**Figure 2:**
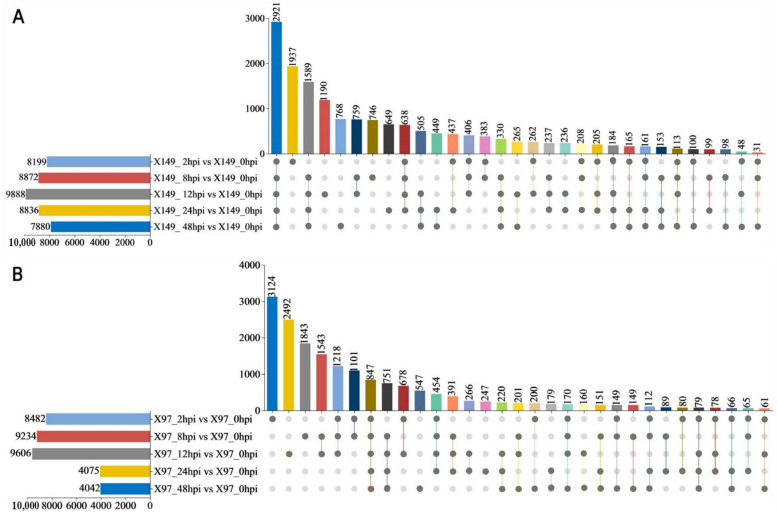

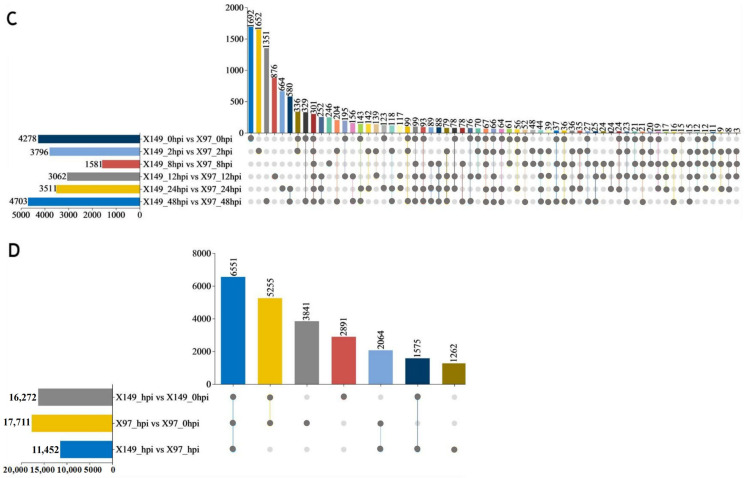
Differential expression analysis (DEGs) in the resistant line X149 and the susceptible line X97. (**A**,**B**) Upset diagram of DEGs after inoculation in X149 (**A**) and X97 (**B**) compared to 0 hpi. (**C**) Upset diagram of DEGs after inoculation between X149 and X97. (**D**) Upset diagram of the DEGs based on (**A**–**C**). The horizontal bar chart on the left represents the comparison groups, different colors represent different comparison groups on A–D figures, respectively. The vertical bar chart represents the shared DEGs numbers of comparison groups, different colors represent different shared DEGs numbers on A-D figures, respectively. In the middle matrix, a single grey point represents the unique DEGs of a comparison group, greater than two connecting gray points represent shared DEGs of different comparison group. X149_hpi includes 0, 2, 8, 12, 24, 48 hpi; X97_hpi includes 0, 2, 8, 12, 24, 48 hpi.

To identify DEGs that responded to SC15 infection, transcriptome comparisons after inoculation (2 hpi vs. 0 hpi, 8 hpi vs. 0 hpi, 12 hpi vs. 0 hpi, 24 hpi vs. 0 hpi, and 48 hpi vs. 0 hpi) were conducted for the resistant material X149 and susceptible material X97, respectively. A total of 16,272 union DEGs (X149_hpi vs. X149_0hpi) were found in the resistant material X149 at five different time points after inoculation compared to 0 hpi (Figure 2A). In the susceptible material X97, a total of 17,711 union DEGs (X97_hpi vs. X97_0hpi) were also identified (Figure 2B). Following analysis of union DEGs in X149 and X97, a total of 22,177 DEGs were identified to respond to SC15 infection (Figure 2D).

To further clarify whether the 22,177 DEGs also differed between the resistant and susceptible materials after SC15 infection, the DEGs were analyzed between the resistant and susceptible materials at 0, 2, 8, 12, 24, and 48 hpi, with 11,452 (X149_hpi vs. X97_hpi) union DEGs identified (Figure 2C). There were 10,190 shared genes in 22,177 DEGs and 11,452 DEGs were found (Figure 2D). These 10,190 DEGs responded to SC15 infection and were differentially expressed in the resistant and susceptible materials, meaning they may be involved in soybean resistance to SC15.

### 3.4. Functional Annotation of DEGs

To evaluate potential function categories of DEGs, 10,190 DEGs were subjected to GO and KEGG annotation analysis. GO annotation analysis revealed that 10,190 DEGs were classified into 50 functional categories, including 22 biological processes, 14 cellular components, and 14 molecular function terms (Table S3). The top eight annotation terms were “binding”, “catalytic activity”, “cell part”, “metabolic process”, “cellular process”, “membrane part”, “organelle”, and “biological regulatory” (Figure S1A). KEGG annotation analysis of 10,190 DEGs revealed that the main enrichment pathways were “carbohydrate metabolism”, “signal transduction”, and “amino acid metabolism” (Figure S1B).

### 3.5. Gene Co-Expression Network Analysis

**Figure 3:**
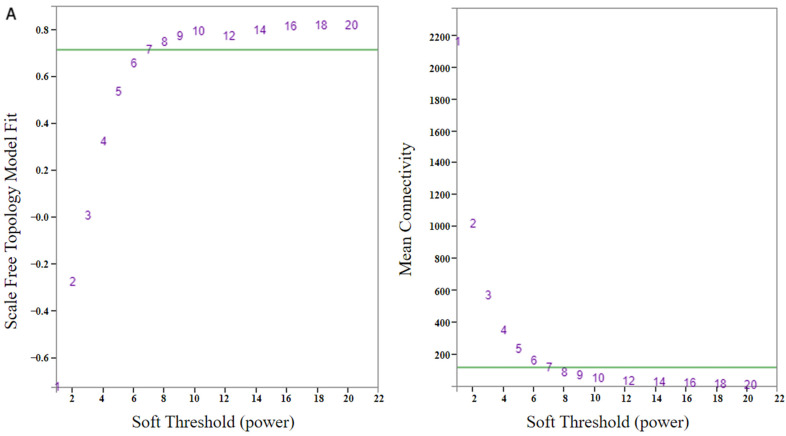

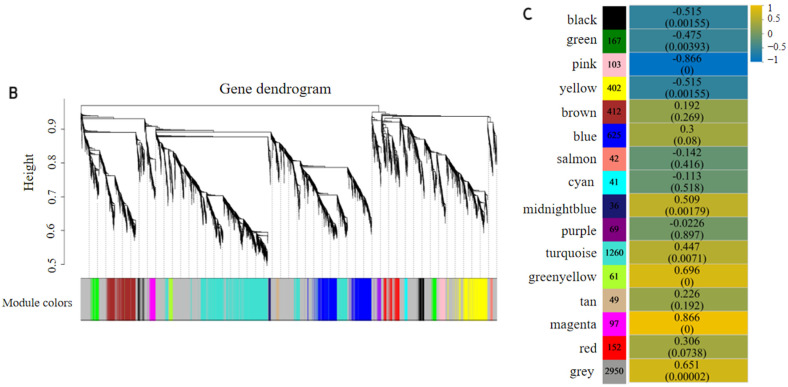
Soft threshold power (β value) determination of WGCNA and modules detection. (**A**) Soft threshold determination. The number corresponding to the green line is the most appropriate β value. (**B**) Gene cluster dendrogram and module colors. (**C**) Correlation between modules and resistance trait. The correlation coefficient is above and the *p* values are shown below each cell.

To avoid the influence of outlier samples, a 36-sample clustering dendrogram was constructed based on the expression of all genes (Figure S2A). Finally, one sample X149_24hpi_1 was eliminated and 35 samples remained for follow-up analysis (Figure S2B). To identify hub modules of resistance in X149 to SC15, co-expressed gene modules were determined using WGCNA based on 6596 DEGs (10,190 DEGs eliminate FPKM < 1 remaining DEGs). For accurate building of the modules, a β value = 7 was chosen (Figure 3A). When the β value was 7, 15 modules with module sizes ranging from 36 to 1260 genes and 2950 genes outside of the 15 modules were classified into a grey module, which was then generated (Figure 3B). Module eigengenes were evaluated in correlation with the resistance phenotype. The results showed that four module eigengenes correlated significantly (*p* < 0.01) positively with SC15 resistance in X149 (Figure 3C): magenta (R = 0.866, *p* = 0), greenyellow (R = 0.696, *p* = 0), midnightblue (R = 0.509, *p* = 0.00179), and turquoise (R = 0.447, *p* = 0.0071) modules. The genes in these correlating modules may positively regulate SC15 resistance. In addition, four module eigengenes had a negative correlation in X149 (Figure 3C): pink (R = −0.886, *p* = 0), black (R = −0.515, *p* = 0.00155), yellow (R = −0.515, *p* = 0.00155), and green (R = −0.475, *p* = 0.00393). The genes in these modules may negatively regulate SC15 resistance. There were 97 genes in the magenta, 61 genes in greenyellow, 36 genes in midnightblue, 1260 genes in turquoise, 103 genes in pink, 130 genes in black, 402 genes in yellow, and 167 genes in green modules (Table S4).

**Figure 4:**
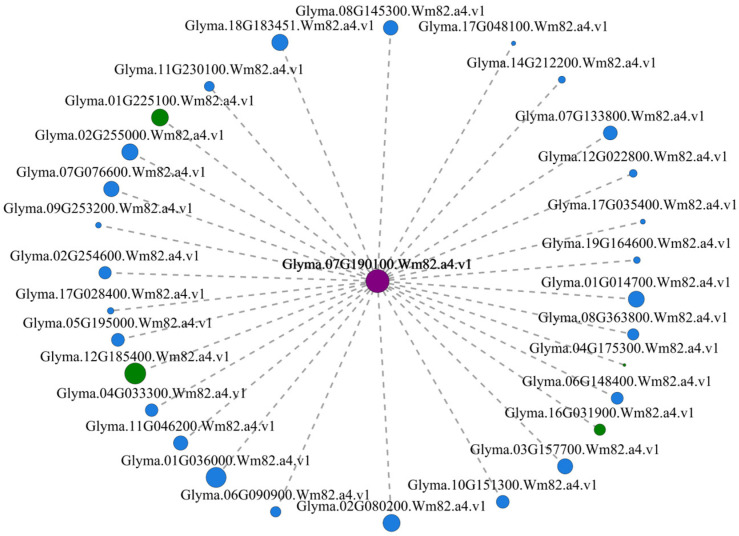
Co-expression networks of the 30 most highly connected hub genes in the eight modules related to resistance. The purple circle represents the core hub gene *Glyma.07G190100*, green circle represents the co-expression hub genes *Glyma.12G185400*, *Glyma.01G255000*, *Glyma.16G031900*, and *Glyma.04G175300*.

To screen hub genes from the eight modules, a co-expression network of eight modules was constructed. The results showed that the 30 most highly connected hub genes belonged to the turquoise module (Figure 4, Table S5). One core hub gene *Glyma.07G190100*, encoding F-box protein, was identified, and its co-expressed hub genes, including gene *Glyma.12G185400* (CMLs), *Glyma.01G225100* (PP2C), *Glyma.16G031900* (WRKY22), and *Glyma.04G175300* (CDPK), were annotated in the plant hormone signal transduction pathway (map04075), plant–pathogen interaction pathway (map04626), and MAPK signaling pathway (map04016), respectively. We speculated five hub genes may be involved in X149 resistance to SC15.

### 3.6. Validation of RNA-Seq Data by RT-qPCR

**Figure 5:**
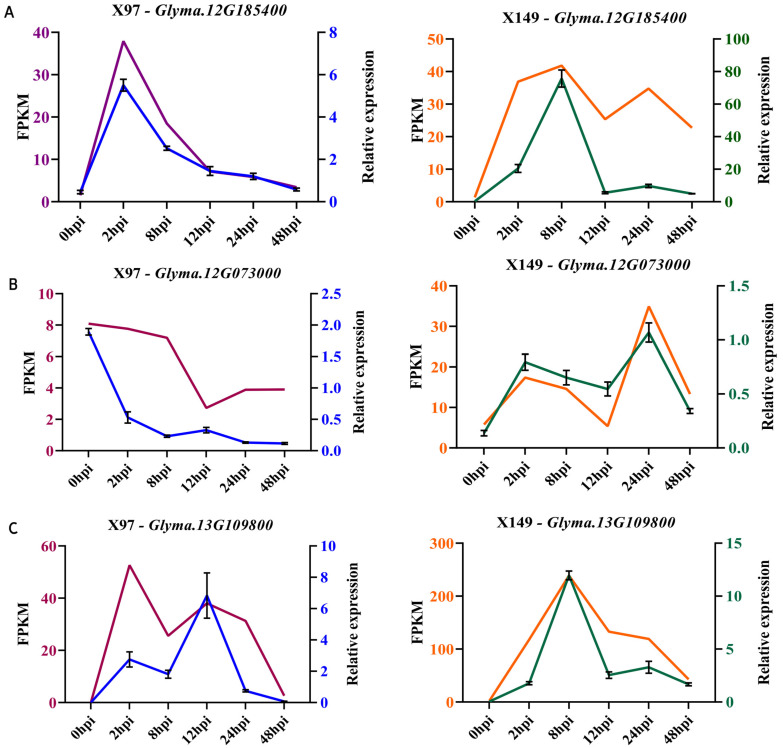

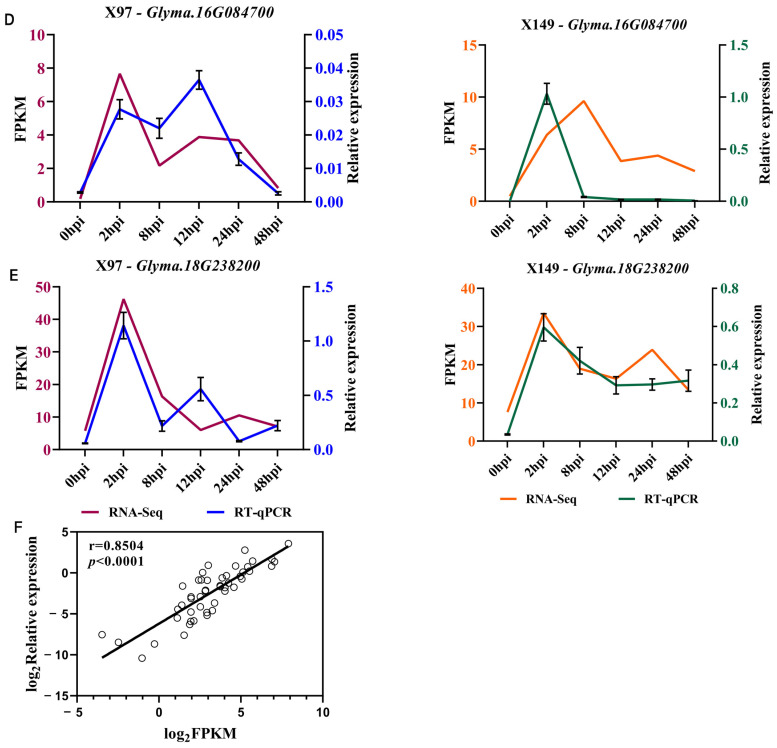
Validation results of RNA–Seq by RT–qRCR for five genes. (**A**–**E**) Expression analysis of the genes *Glyma.12G185400*, *Glyma.12G07300*, *Glyma.13G109800*, *Glyma.16G084700*, and *Glyma.18G238200* in the susceptible line X97 (left) and the resistant line X149 (right). The left vertical axis indicates the FPKM value obtained via RNA-Seq, and the right vertical axis indicates the relative expression level determined via RT-qPCR. (**F**) Correlation analysis between RT-qPCR and RNA-seq data. r is the correlation coefficients; *p* represents a significant correlation.

To validate the level of RNA-Seq expression, five genes related to resistance were randomly selected from the above eight modules for RT-qPCR at the six time points in X97 and X149. RT-qPCR expression analysis revealed that the overall expression trends were consistent with the RNA-Seq data for all genes, and the expression levels differed (Figure 5A–E). The statistical analysis also showed very good correspondence (correlation coefficient of 0.8025) among the results of the RT-qPCR and RNA-seq analysis (Figure 5F). Considering all these findings, we believe that the RNA-Seq data are reliable.

### 3.7. KEGG Pathway Enrichment Analysis of DEGs of Eight Modules

**Figure 6:**
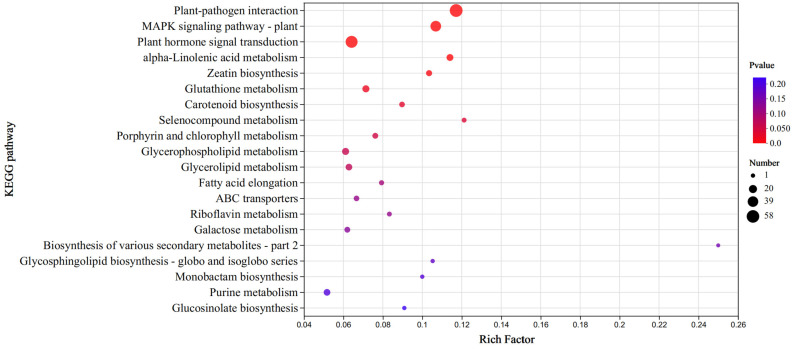
KEGG enrichment analysis of 2256 DEGs. The x-axis indicates the ratio of the number of DEGs in the pathway to all DEGs. The y-axis indicates the KEGG pathway.

To further explore the regulatory pathway for X149 resistance to SC15, KEGG pathway enrichment analysis was performed based on the 2256 DEGs of eight modules. The results showed that a total of 20 pathways were enriched (Figure 6). The top three pathways were significantly clustered into pathogen-resistance-related pathways, such as “Plant–pathogen interaction (map04626)”, “MAPK signaling pathway (map04016)”and “Plant hormone signal transduction (map04075)”. These results could provide a reference for further screening of resistance-related key pathways in X149.

### 3.8. Key Regulatory Pathways in the Plant–Pathogen Interaction Pathway, MAPK Signaling Pathway, and Plant Hormone Signal Transduction Pathway Analysis

**Figure 7:**
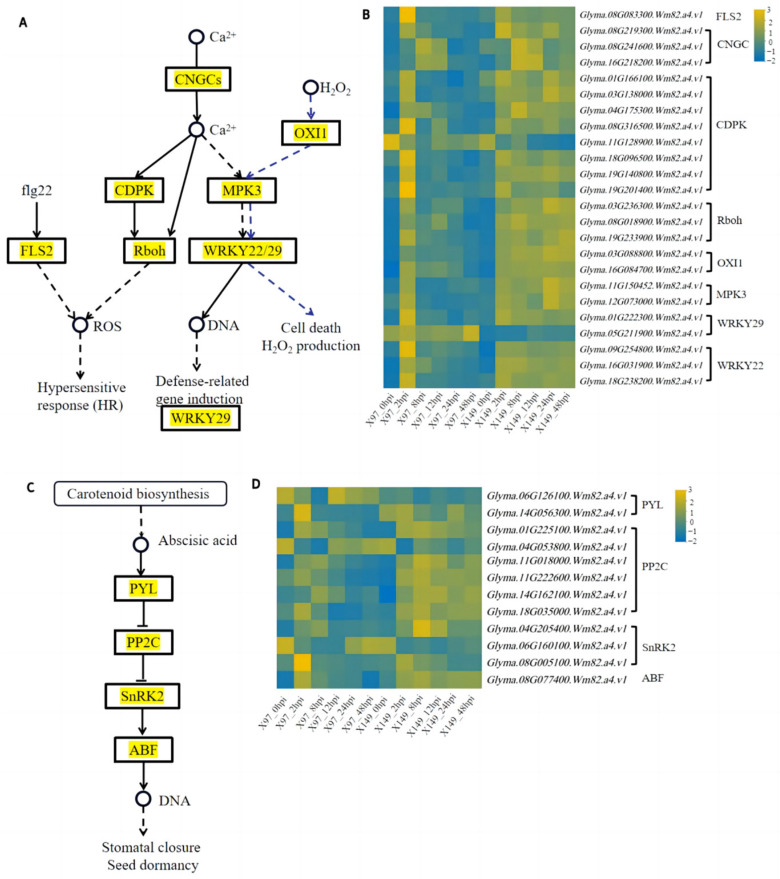
Key pathways information of the DEGs. (**A**) Key pathways of DEGs in the plant–pathogen interaction pathway and MAPK signaling pathway. The black arrow shows the flg22 and Ca^2+^ regulation pathway in the plant–pathogen interaction pathway, the purple arrow shows the H_2_O_2_ regulation pathway in the MAPK signaling pathway. The yellow parts indicate the presence of the corresponding DEGs. (**B**) Heatmaps of flg22, Ca^2+^ and H_2_O_2_ regulation pathway DEGs. The log_2_FPKM values are colored. Yellow, up-regulated; blue, down-regulated. (**C**) Key pathways of DEGs in plant hormone signal transduction. The black arrow shows the ABA regulation pathway. The yellow parts indicate the presence of the corresponding DEGs. (**D**) Heatmaps of ABA regulation pathway DEGs. The log_2_FPKM values are colored. Yellow, up-regulated; blue, down-regulated.

According to the KEGG pathway enrichment analysis results, 58 and 39 DEGs were involved in the plant–pathogen interaction pathway and MAPK signaling pathway, respectively (Table S6). The genes were clustered into pathways, which revealed that the complete Ca^2+^ and flg22 (well-conserved N-terminal region of flagellin) regulatory pathways were found in the plant–pathogen interaction pathway, and the complete H_2_O_2_ regulatory pathway was found in the MAPK signaling pathway (Figure 7A). These three complete regulatory pathways were considered most likely to be involved in SC15 resistance. Mitogen-activated protein kinase (MPK3) and WRKY transcription factors (WRKY22/29) were found in both the Ca^2^+ and H_2_O_2_ regulatory pathways (Figure 7A).

In the flg22 regulatory pathway, *Glyma.08G083300*, which encodes leucine-rich repeat (LRR) receptor-like serine/threonine-protein kinase FLS2 (FLS2), exhibited lower expression at 0 hpi and up-regulated expression at 2 hpi in the resistance line X149 and susceptible line X97, followed by higher expression in X149 than in X97 overall (Figure 7B). In the Ca^2+^ regulatory pathway, *Glyma.08G21930*0, *Glyma.08G241600*, and *Glyma.16G218200* encode cyclic nucleotide-gated channels (CNGCs); seven DEGs (*Glyma.01G166100*, *Glyma.03G138000*, *Glyma.04G175300*, *Glyma.08G316500*, *Glyma.18G096500*, *Glyma.19G140800*, and *Glyma.19G201400*) encode CDPK; three DEGs (*Glyma.03G236300*, *Glyma.08G018900*, and *Glyma.19G233900*) encode the respiratory burst oxidase homologue Rboh; two DEGs encode MPK3 (*Glyma.11G150452* and *Glyma.12G073000*); *Glyma.01G222300* encodes WRKY29; and three DEGs (*Glyma.09G254800*, *Glyma.16G031900*, and *Glyma.18G238200*) encode WRKY22, which also showed lower expression at 0 hpi and was up-regulated at 2 hpi in resistance line X149 and susceptible line X97, followed by higher expression in X149 than in X97 as a whole (Figure 7B). In addition, *Glyma.11G128900*, which encodes CDPK, and *Glyma.05G211900*, which encodes WRKY29, exhibited lower expression in X149 than in X97 (Figure 7B). In the H_2_O_2_ regulatory pathway, *Glyma.03G088800* and *Glyma.16G084700*, which encode OXI1, exhibited lower expression at 0 hpi and up-regulated expression at 2 hpi in the resistance line X149 and susceptible line X97, followed by higher expression in X149 than in X97 overall (Figure 7B). The expression data of DEGs related to MPK3, WRKY29, and WRKY22 were consistent with the Ca^2+^ regulatory pathway. These results indicated that the expression of all 24 DEGs was induced by SC15 infection and that there were differences in expression of X149 and X97. We speculated that these genes may be involved in soybean resistance to SC15. The resistant line X149 may resist SC15 infection via Ca^2+^, flg22, and H_2_O_2_ regulatory pathways.

The plant hormone signal transduction pathway included 50 DEGs (Table S6) and the complete ABA regulatory pathway was covered with 12 DEGs (Figure 7C). Overall, *Glyma.06G126100*, which encodes PYRABACTIN resistance/PYR-like (PYL), exhibited higher expression in X97 than in X149, while *Glyma.14G056300* was the opposite (Figure 7D). Among the six DEGs, *Glyma.04G053800* (PP2C) was more highly expressed in X97 than in X149 as a whole, and the other five DEGs (*Glyma.01G225100*, *Glyma.11G018000*, *Glyma.11G222600*, *Glyma.14G162100*, and *Glyma.18G035000*) were more highly expressed in X149 than in X97 as a whole (Figure 7D). *Glyma.04G205400*, *Glyma.06G160100*, and *Glyma.08G005100* encode SnRK2 (sucrose non-fermenting 1-related protein kinase 2), and the expression of *Glyma.04G205400* and *Glyma.08G005100* were greater in X149 than in X97 overall, while the opposite trend was observed for *Glyma.06G160100* (Figure 7D). The expression of *Glyma.08G077400*, which encodes ABA-responsive element binding factors (ABF), was greater in X149 than in X97 overall (Figure 7D). These results showed that the expression of 12 DEGs in the ABA regulatory pathway was induced by SC15 infection, and differences in the expression of these genes were detected between X149 and X97. We speculate that these genes may be involved in soybean resistance to SC15. The resistant line X149 may resist SC15 infection via the ABA regulatory pathway.

### 3.9. Assay of SOD, CAT, and POD Activitives and H_2_O_2_ Level in X149

**Figure 8:**
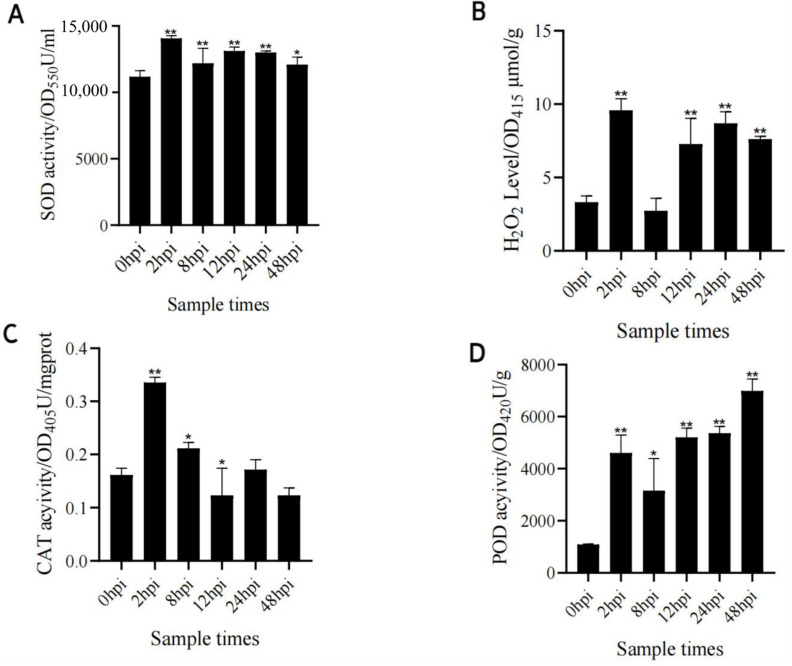
Detection of the SOD activity (**A**), H_2_O_2_ content (**B**), CAT activity (**C**), and POD activity (**D**) at different time points in resistance line X149. * *p* < 0.05 and ** *p* < 0.01 indicate significant and extremely significant differences.

**Figure 9:**
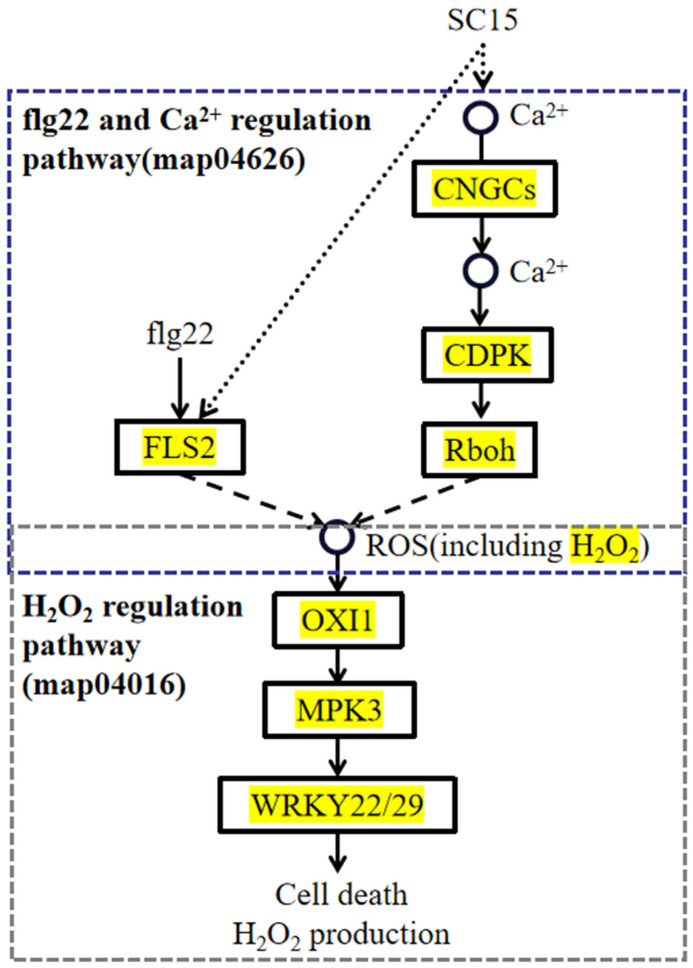
Diagram of the H_2_O_2_ regulation pathway, which may be dependent on ROS generation of flg22 and the Ca^2+^ regulation pathway in SC15-infected soybeans. Purple dotted box refers to flg22 and the Ca^2+^ regulation pathway, grey dotted box refers to the H_2_O_2_ regulation pathway. The yellow parts indicate the presence of the corresponding DEGs.

To confirm that H_2_O_2_ generation occurred after SC5 infection, SOD (which catalyzes O^2•−^ dismutation to H_2_O_2_), H_2_O_2_ content, and CAT and POD activities (which catalyze the degradation of H_2_O_2_) were measured in the resistant line X149. Overall, the results showed that SOD activity (Figure 8A), H_2_O_2_ content (Figure 8B), and POD activity (Figure 8D) were significantly up-regulated compared with those at 0 hpi, and CAT activity (Figure 8C) was significantly up-regulated at 2 hpi–12 hpi. These findings indicated that H_2_O_2_ was indeed generated in the resistant line X149 in response to SC15 infection. In addition, the H_2_O_2_ was related to ROS, so we speculate that the H_2_O_2_ regulatory pathway may depend on flg22 and Ca^2+^ regulatory pathway to induce ROS generation in the resistance of X149 to SC15 (Figure 9).

## 4. Discussion

Conventional transcriptome analysis has been applied to the analysis of molecular regulatory networks of soybean resistance to SMV. Although the plant–pathogen interaction pathway, MAPK signaling pathway, and plant hormone signal transduction pathway have been showed to respond to SMV infection, this response was mainly due to the role of some individual genes in the pathways [35,36,37,38,39]. The WGCNA method can identify the relationship between module eigengenes and hub genes with sample traits. In this study, we used the WGCNA method to obtain resistance genes, and then resistance pathways were screened by KEGG pathway enrichment analysis. In total, four complete pathways, including flg22, Ca^2+^, H_2_O_2_, and ABA regulatory pathways, and five hub genes were identified. This realized the transformation from macroscopic pathways to microscopic complete pathways. We also found that there is a cascade relationship between complete pathways by measuring H_2_O_2_.

### 4.1. flg22 Regulatory Pathway as Crucial SC15-Responsive Pathway

When plants are infected with pathogens, PRR-triggered immunity (PTI) is activated by pattern recognition receptors (PRRs) that recognize pathogen- or microbe-associated molecular patterns (PAMPs or MAMPs) or damage-associated molecular patterns (DAMPs) [62]. PTI can trigger various responses, including rapid generation of reactive oxygen species (ROS), activation of MAPKs, and expression of immune-related genes [62,63]. PTI against bacteria depends on the recognition of the flagellin-derived MAMP flagellin 22 (flg22) by the receptor kinase FLS2 [63,64]. It has been reported that the *tomato yellow leaf curl virus* (TYLCV) C4 protein can also interact with FLS2 of Arabidopsis [65]. This means that the recognition of the virus and FLS2 can trigger PTI. Coat protein (CP) of *Tobacco mosaic virus* (TMV) can elicit ROS bursts in tobacco [66]. Silencing of movement implicated that protein P6 from the *Cauliflower mosaic virus* (CaMV) could suppress PAMP-induced ROS generation in Arabidopsis [63]. In the present study, the flg22 regulatory pathway was investigated. The expression level of the FLS2 gene in the resistant line 149 was greater than that in the susceptible line 97 after SC15 infection. The flg22 regulatory pathway mainly triggers the rapid generation of ROS according to the KEGG pathway enrichment results. In conclusion, flg22 regulatory pathway may positively affect X149 resistance to SC15 through the production of ROS.

### 4.2. Ca^2+^ and H_2_O_2_ Regulatory Pathways as Crucial SC15-Responsive Pathways

Ca^2+^ signaling is linked to the activation of plant defense reactions [67]. The concentration of intracellular Ca^2+^ increases immediately when PAMPs are present, and Ca^2+^ influx is influenced by cyclic nucleotide-gated channels (CNGCs) [67]. CDPKs are Ca^2+^ sensors and transducers, and their main function is related to biotic and abiotic stresses in plants [68,69]. In Arabidopsis, the N-terminal EF-hands of RbohF and RbohD were bound directly by Ca^2+^ and were also activated at the main sites by CDPK, and then RbohF and RbohD regulated ROS generation [64]. *AtRbohF* is probably involved in resistance to *Magnaporthe oryzae* in Arabidopsis [70]. When RbohD was knocked out in *Arabidopsis thaliana*, the *Turnip mosaic virus* (TuMV) infection was promoted in *rbohD* plants, suggesting that RbohD plays a role in TuMV resistance [71]. In this study, DEGs related to CNGCs, CDPK, and Rboh in the Ca^2+^ regulatory pathway were all enriched, and the expression of most DEGs was higher in X149 than X97. These findings suggest that Ca^2+^ regulatory pathway may positively influence X149 resistance to SC15 through the production of ROS. But it has been reported that Ca^2+^ regulatory pathway showed a negative influence on SC15 resistance in resistance line Kefeng 1 [39]. This difference may be caused by the different genetic backgrounds of the soybean resources and samples times. 

In the H_2_O_2_ regulatory pathway, the mitogen-activated protein kinase (MAPK) MPK3 is activated by OXI1 and has been reported to regulate the defense response pathway against pathogens via WRKY22 and WRKY29 [72,73]. MPK3 in resistance cultivar L29 showed hallmarks of *Rsv3*-mediated extreme resistance (ER) against SMV-G5H [74]. WRKY22 can regulate programmed cell death (PCD) to restrict the spread of the pathogen Pst [75]. WRKY29 was induced in Arabidopsis in response to *Fusarium graminearum* infection [76]. In this study, DEGs related to OXI1, MPK3, and WRKY22/29 in the H_2_O_2_ regulatory pathway were all enriched, and the expression of most DEGs in X149 was greater than that in the susceptible line 97 after SC15 infection. This indicates that the H_2_O_2_ regulatory pathway positively influenced X149 resistance to SC15. In addition, MPK3 and WRKY22/29 were also enriched in the Ca^2+^ regulatory pathway. These findings suggest that there may be potential cross-talking between the Ca^2+^ regulatory pathway and H_2_O_2_ regulatory pathway.

### 4.3. ABA Regulatory Pathway as Crucial SC15-Responsive Pathway

ABA, as a signal-regulated signal transduction network, is a plant hormone that can be synthesized in higher plants by carotenoid biosynthesis and plays an important role in plant responses to abiotic and biotic stresses [77,78]. In the present study, DEGs related to PYL, PP2C, SnRK2, and ABF in the ABA regulatory pathway were completely enriched, and the expression of most DEGs was higher in X149 than X97. The soybean PP2C-type gene *GmPP2C3a* was proven to mediate the extreme resistance of soybeans to SMV *Rsv3* and was up-regulated in the resistant line after infection with SC3 [37,79]. Similarly, the expression of *PtrSnRK2.4* and *PtrABF2* in trifoliate orange increased in response to drought stress [80]. We believe that the resistance of X149 to SC15 may depend on the positive regulation of the ABA regulatory pathway.

### 4.4. Candidate SC15-Resistance Hub Gene Mining

Hub genes play a crucial role in interpreting plant phenotypes. Three hub genes were found to belong to above three pathways, *Glyma.01G225100* (PP2C), located in the ABA regulatory pathway; *Glyma.16G031900* (WRKY22), located in the H_2_O_2_ and Ca^2+^ regulatory pathways; and *Glyma.04G175300* (CDPK), located in the Ca^2+^ regulatory pathway. These suggest that it is reliable to use these three pathways as SC15-resistance-related pathways. Further, *Glyma.12G185400* (CMLs) , another sensor of Ca^2+^ that acts as a hub gene, was identified in this study, and its expression was higher in X149 than in X97 (Figure 5A). Three CML genes (*Glyma03g28650*, *Glyma19g31395*, and *Glyma11g33790*) with down-regulated expression in the susceptible line NN1138-2 were very likely to promote SMV infection [37]. Overexpression of a CML named rgs-CaM can enhance resistance to *cucumber mosaic virus* (CMV) [81]. These showed that gene *Glyma.12G185400* positively regulated SC15 resistance in X149.

One core gene, *Glyma.07G190100*, encoding F-box protein, was also identified. The F-box-domain-containing proteins are a superfamily found in eukaryotic cells, including plants, yeast, and mammals. Proteins encoded by plant F-box genes play a variety of roles in developmental processes, including plant hormonal signal transduction, secondary metabolism, and responses to both biotic and abiotic stresses, etc. [82]. The F-box gene *OsDRF1* was induced by ABA treatment and rice blast fungus *Magnaporthe grisea* infection. Overexpressing *OsDRF1* in tobacco plants leads to enhanced disease resistance against *tomato mosaic virus* (ToMV) and *Pseudomonas syringae* pv. *Tabaci* [83]. The expression of F-box gene *TaFBA1* of wheat is induced by oxidative stress. Overexpression of *TaFBA1* in tobacco plants enhanced oxidative stress tolerance through lower ROS accumulation and higher activities of antioxidant enzymes, including SOD, CAT, and POD, observed in the transgenic plants than those in WT [84]. This manifested F-box gene was related to ABA treatment and ROS accumulation. *Glyma.07G190100* and other hub genes all belonged to turquoise module and they owned similar expression profiles. We conjectured that gene *Glyma.07G190100* positively regulated X149 resistance to SC15.

### 4.5. Change of Antioxidant Enzyme Activities and H_2_O_2_ Content in X149 under SC15 Infection

ROS are unavoidable by-products of metabolism [44]. In infected plants, the accumulation of ROS has a dual role. It can promote programmed cell death (PCD) in pathogenic cells and restrict invading pathogens to the infected places [85]. But excess production of ROS can damage lipids, proteins, and DNA [86]. So, the production and elimination of ROS must be tightly regulated. Rbohs, plant NADPH oxidases, are responsible for this ROS generation [71]. The localization and distribution of RbohD differed in potato virus Y (PVY^NTN^)-compatible potatoes and PVY^NTN^-incompatible potatoes. RbohD localization, followed by H_2_O_2_ detection, was concentrated in the apoplast in the higher-resistance cultivar, while distribution of RbohD was concentrated in the plant cell organelles in the lower-resistance cultivar. Further, high induction of RbohD, found in PVY^NTN^-compatible potatoes, was associated with necrotization [87]. Scavenging or detoxification of the excess ROS can be carried out through the enzymatic antioxidants encompassing SOD, POD, and CAT [85]. In an antioxidant defense mechanism, SOD catalyzes O^2•−^dismutation to H_2_O_2_, which is later reduced to water and molecular oxygen through CAT, POD, and ascorbate peroxidase (APX) enzymes catalyzing [88]. In the present study, H_2_O_2_ levels and SOD activity were up-regulated after SC15 infection. These results indicated that ROS, including H_2_O_2_, were generated in X149 after SC15 infection. Meanwhile, the increased activities of CAT and POD in the resistant line X149 were found after SC15 infection and can reduce the damage of ROS to cells.

H_2_O_2_ content in resistant soybean cultivar RN-9 was also increased after SC15 infection [2]. Abundant H_2_O_2_ was produced on the cell wall and cell membrane in soybean resistance cultivar Jidou 7 after SMV strain N3 infection, and could increase callose accumulation [89]. According to regulatory pathways study, we found that flg22 and the Ca^2+^ regulatory pathway can induce ROS generation. The H_2_O_2_ regulatory pathway was an important SC15-responsive pathway. H_2_O_2_ levels were indeed induced to accumulate in the resistant line X149 when infected with SC15. In summary, we believe that the H_2_O_2_ regulatory pathway may depend on flg22 and the Ca^2+^ regulatory pathway to induce ROS generation in the resistance of X149 to SC15.

## 5. Conclusions

In this study, a co-expression network of weighted genes associated with X149 SC15-resistant traits was constructed to identify 15 target modules. Eight target gene modules were significantly associated with SC15 infection. KEGG pathway enrichment analysis showed 2256 DEGs of eight modules enriched the top three pathways, which were pathogen-resistance-related pathways, MAPK signaling pathway, and plant hormone signal transduction pathway. In total, 147 DEGs were included in top three pathways, and among these, 36 DEGs could annotate four complete pathways, including flg22, Ca^2+^, H_2_O_2_, and ABA regulatory pathways. The expression levels of 33 DEGs out of 36 DEGs were higher in resistant line X149 than in susceptible line X97. Four complete regulatory pathways were hypothesized to positively regulate soybean resistance. Meanwhile, the 30 most highly connected hub genes belonging to the turquoise module were identified. Among three hub genes, *Glyma.01G225100* (PP2C), *Glyma.04G175300* (CDPK), and *Glyma.16G031900* (WRKY22) were annotated in the ABA, Ca^2+^, and H_2_O_2_ regulatory pathways and their expressions in X149 were greater than in X97. This can further verify the accuracy of the resistance regulatory pathways. Further, other hub genes, *Glyma.12G185400* (CMLs) and F-box protein gene *Glyma.07G190100*, were reported to be involved in biotic and abiotic stresses and their expressions were higher in resistant line X149 than in susceptible line X97. These two hub genes were thought to positively regulate soybean resistance. We also found H_2_O_2_ levels were up-regulated after SC15 infection. We believe that the H_2_O_2_ regulatory pathway may depend on flg22 and the Ca^2+^ regulatory pathway to induce ROS generation. The results of this study benefit further understanding of the molecular mechanisms of SC15 resistance and provide a new gene resource for future SC15 resistance breeding in soybean.

## Data Availability

The original contributions presented in the study are included in the article/supplementary material.

## Supplementary Materials

The following supporting information can be downloaded at: https://www.mdpi.com/article/10.3390/genes15050566/s1, Figure S1: Functional annotation of 10,190 DEGs. (A) GO annotations analysis. (B) KEGG annotations analysis; Figure S2: Clustering dendrograms of samples. (A) Dendrogram of all 36 samples. The one outlier sample is written in red fonts. (B) Dendrogram of 35 samples after removing the one outlier. Table S1: Primers used for RT-qPCR; Table S2: A statistical summary for all RNA-Seq samples; Table S3: GO annotation analysis of 10,190 DEGs; Table S4: Information of 2256 DEGs in 8 modules related to SC15 resistance; Table S5: Information of the most highly connected 30 hub genes; Table S6: DEGs ID information of three major resistance-related pathways.
