# Supplementary material for: WGCNA Reveals Hub Genes and Key Gene Regulatory Pathways of the Response of Soybean to Infection by Soybean mosaic virus
Source: Genes (Basel). 2024 Apr 27;15(5):566. doi: 10.3390/genes15050566 (PMC11120672; doi:10.3390/genes15050566)
Supplement: Supplementary file 1 [file genes-15-00566-s001.zip › Supplementary Figure .pdf]

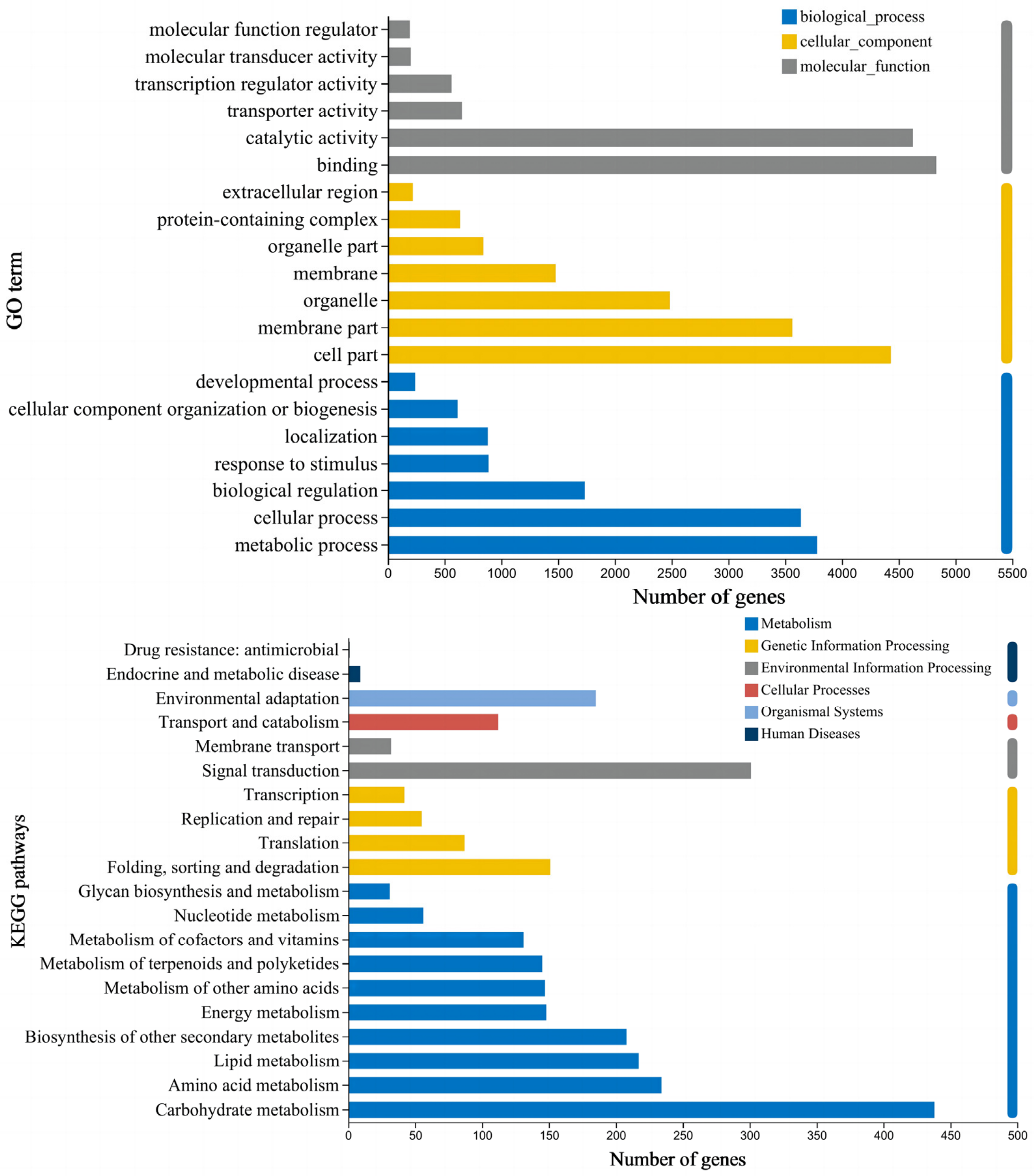

Figure S1 Functional annotation of 10,190 DEGs. (A) GO annotations analysis. (B) KEGG annotations analysis.

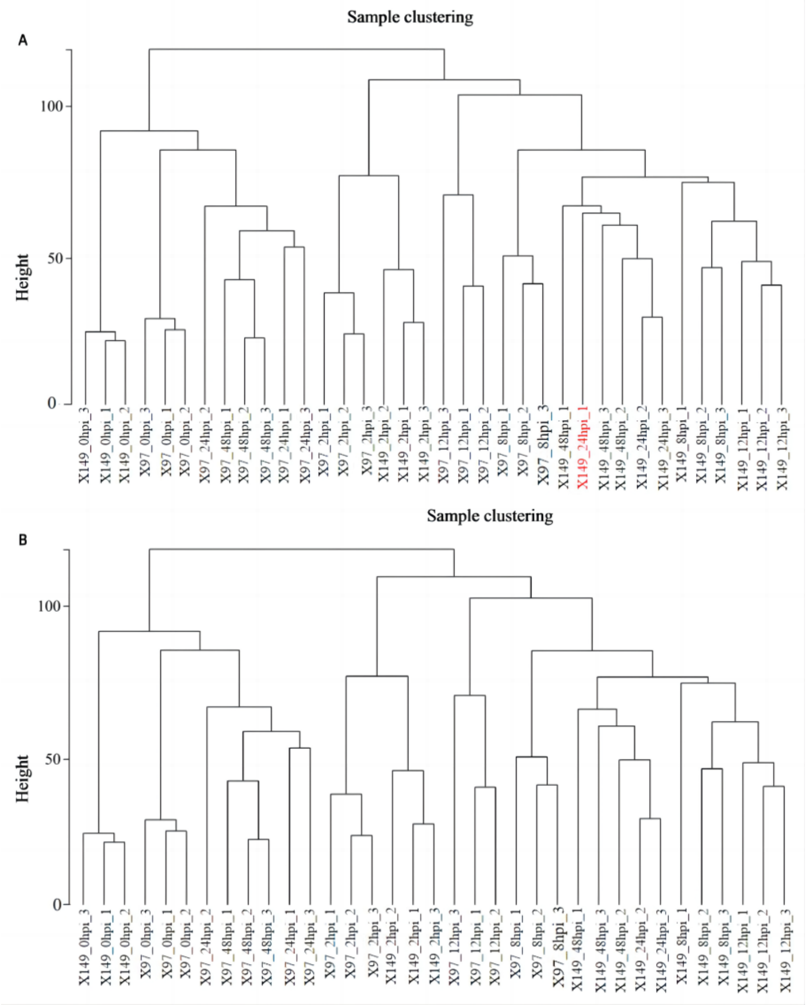

Figure S2 Clustering dendrograms of samples. (A) Dendrogram of all 36 samples. The one outlier sample is written in red fonts. (B) Dendrogram of 35 samples after removing the one outliers.
